# Esophagogastroduodenal pneumatosis with subsequent pneumoporta and intramural duodenal hematoma after endoscopic hemostasis: a case report

**DOI:** 10.1186/s12876-015-0351-x

**Published:** 2015-09-25

**Authors:** Wei-Cheng Huang, Chih-Hsin Lee, Fat-Moon Suk

**Affiliations:** 1 Divisions of Gastroenterology, Wan Fang Hospital, Taipei Medical University, No. 111, Section 3, Hsing Long Road, Taipei, 116 Taiwan; 2 Divisions of Pulmonology, Department of Internal Medicine, Wan Fang Hospital, Taipei Medical University, Taipei, Taiwan; 3 Department of Internal Medicine, School of Medicine, College of Medicine, Taipei Medical University, Taipei, Taiwan

**Keywords:** Pneumatosis intestinalis, Intramural hematoma, Hemostasis, Duodenum, Endoscopy

## Abstract

**Background:**

Esophagogastroduodenal pneumatosis is the presence of air in esophagus, stomach, and duodenum simultaneously, which have never been described in the literature. Intramural duodenal hematoma (IDH) rarely occurs after endoscopic intervention. The diagnosis and treatment in both conditions are great challenge in daily practice.

**Case presentation:**

A 70-year-old male patient, who had been taking warfarin for artificial valve replacement, developed IDH and esophagogastroduodenal pneumatosis after endoscopic hemostasis for duodenal ulcer bleeding. Initially, he had abdominal pain, gastrointestinal bleeding and hypotension. Later, he was found to have acute pancreatitis, biliary obstruction, gastric outlet obstruction and rapid decline of hemoglobin also ensued. The intramural duodenal hematoma and critical condition resolved spontaneously after conservative medical treatment.

**Conclusion:**

Based on this case report, we suggest that intramural duodenal hematoma should be considered if a patient has the tetrad of pancreatitis, biliary obstruction, gastric outlet obstruction and rapid decline of hemoglobin after an endoscopic intervention. Those patients could be treated conservatively. But, surgery should be considered if the diseases progress or complications persist.

## Background

Pneumatosis intestinalis (PI), the presence of gas in the wall of the gastrointestinal tract, has been observed from esophagus to rectum. Some patients with PI carry a benign clinical course, and the gas can be absorbed spontaneously in most instances. But, PI can also lead to a fetal outcome in some patients [1]. To our knowledge, simultaneous pneumatosis of the esophagus, stomach and duodenum has never been reported.

Intramural duodenal hematoma (IDH) is a rare condition, and it occurs mostly after a blunt abdominal trauma [2]. The IDH after endoscopic intervention is even rare. Herein, we report a case of a patient who developed both esophagogastroduodenal pneumatosis and IDH after an endoscopic hemostasis for treating duodenal ulcer bleeding.

## Case presentation

A 70-year-old male patient presented himself to the emergency department due to having had productive cough for one week. He has a past history of type 2 diabetes mellitus, stage III chronic kidney disease, hypertension, old pulmonary tuberculosis, and poliomyelitis. He also received a mechanical valve replacement for severe tricuspid regurgitation and has been taking warfarin for 10 years. He was admitted with the diagnosis of community-acquired pneumonia, and received amoxicillin/clavulanic acid.

**Fig. 1:**
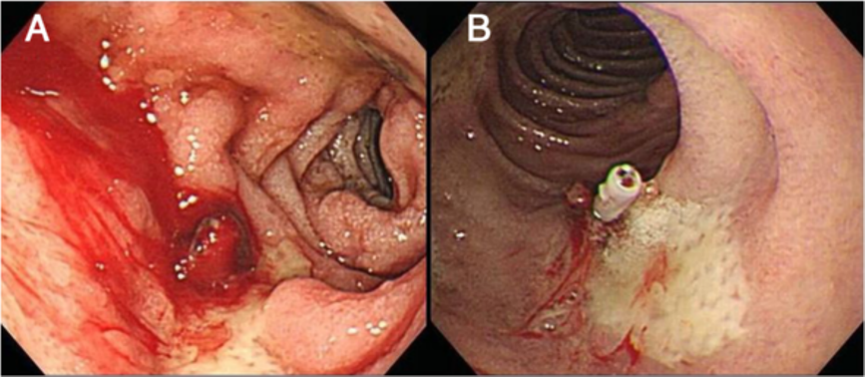
**a** Upper gastrointestinal panendoscopy showed an exposed vessel with active oozing on the ulcer at the inferior wall of the duodenum. **b** The bleeding was stopped after hemoclipping and epinephrine injection. No immediately intramural lesion was found after the procedure

On the second day after admission, the patient developed massive upper gastrointestinal bleeding with a remarkable decrease of hemoglobin from 11.1 g/dL to 7.6 g/dL. He had a profound coagulopathy with prothrombin time (PT) of 43.4 s, international normalized ratio (INR) of 4.07, and activated partial thromboplastin time (aPTT) of 63.2 s. The platelet count was 252,000/μL. Warfarin was discontinued. He received blood component therapy and Vitamin K1 to correct anemia and coagulopathy. He also received intravenous esomeprazole. The panendoscopic examination showed an ulcer on the duodenal bulb with an active oozing vessel on the ulcer crater (Fig. 1-a). Hemostasis was performed with epinephrine injection and hemoclipping (Fig. 1-b).

**Fig. 2:**
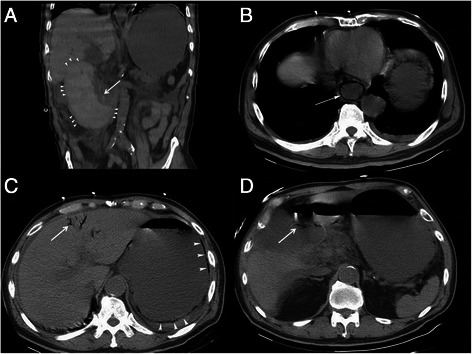
Abdomen and pelvis computed tomography showed a 14.4 cm × 7 cm mass lesion (**a**, arrowhead) at the lateral side of duodenal second portion and caused lumen narrowing (**a**, arrow). The distended stomach is suggested to have gastric outlet obstruction. Pneumatosis was found at esophagus (**b**, arrow), stomach (**c**, arrowhead) and the bulb (**d**, arrow). The inflated air also entered the portal system (**c**, arrow)

The patient had abdominal pain, gastrointestinal bleeding and hypotension one day after the endoscopic procedure. Hemoglobin was further dropped to 5.8 g/dL, and coagulopathy was worsened with PT of 60.4 s, INR of 5.69, and aPTT of 60.1 s despite of aggressive component therapy. Total serum bilirubin level was rapidly elevated from 1.68 mg/dL to 6.00 mg/dL. Acute pancreatitis was also suspected with a lipase level of 3743 IU/L. Computed tomography of the abdomen showed a 14.4 cm × 7 cm intramural hematoma at the second portion of duodenum (Fig. 2-a). The stomach was distended which indicated gastric outlet obstruction. He also had air retention in the portal vein and wall of esophagus, stomach and bulb (Fig. 2- b to d).

**Fig. 3:**
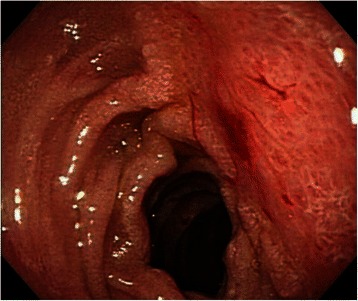
Follow-up panendoscopy showed resolution of intramural duodenal hematoma, which was associated with swelling of mucosa and diminished villi on the lateral side of duodenum

The patient developed respiratory failure and shock, and he received support with mechanical ventilation. His bleeding tendency was corrected by vitamin K1 and blood component therapy. Hemodynamic instability was promptly resolved within two days. Abdominal ultrasonography taken six days later showed the finding of complete resolution of the duodenal hematoma, and was confirmed with endoscopy two weeks later (Fig. 3). But, his renal function was progressively worsened, and the patient and his family declined hemodialysis therapy. The patient died of renal failure on the 40th day after admission.

Depending on the involved site of gastrointestinal tract, clinical presentation of PI is usually nonspecific, varying from chest pain, diarrhea, constipation, abdominal pain, abdominal distension, nausea, vomiting, to gastrointestinal bleeding, or even no symptom [1, 3, 4].

Gastric pneumatosis has been described in various circumstances, including patients who have hemorrhagic radiation gastritis after Argon plasma coagulation [5], gastric ulcer [6], corrosive injury of stomach, status post endoscopy, gastric outlet obstruction, duodenal obstruction, nasogastric tube placement [3], hepatectomy with vascular reconstruction, obstructive pulmonary disease with bleb rupture [7], and high-dose dexamethasone therapy [8]. It can be divided into two categories: first, gastric emphysema, implying benign condition and usually being managed by conservative treatment; and second, emphysematous gastritis, suggesting emergent condition, and surgical intervention being critical for life-saving. Mclaughlin *et al.* have proposed to use the term gangrenous PI/nongangrenous PI instead of emphysematous gastritis/gastric emphysema, to avoid misleading [7].

IDH is also a rare condition that mostly occurs after a blunt abdominal trauma because that the duodenum has rich submucosal vascular supply and is fixed in retroperitoneum [9]. IDH has also been reported as clinical finding associated with anticoagulant therapy, blood dyscrasia, pancreatic disease, collagen vascular disease [10], and diagnostic/therapeutic endoscopy [11] such as endoscopic retrograde cholangiopancreatography with sphincterotomy and biliary stone retrieval [12]. It tends to occur in patients with liver cirrhosis, coagulopathy and hemodialysis especially [11]. IDH is usually confined from the first portion to the second portion of the duodenum due to the barrier of pylorus and ligament of Treitz. Typical symptoms of IDH include epigastric pain, vomiting, and hematochezia. Acute pancreatitis is the most frequent comorbidity in those patients [13]. Thandassery *et al.* reported a rare case in a patient who developed intramural duodenal hematoma after endoscopic retrograde cholangiopancreatogram (ERCP) with sphincterotomy and biliary stone extraction with subsequent of acute pancreatitis, biliary and gastric outlet obstruction after ERCP [12]. Our patient also had the triad of above symptoms. We also observed rapid decline of hemoglobin level in our patient and this observation has been described in other case reports [10, 14]. We suggest that the patients should be suspected to have IHD if they have the tetrad of acute pancreatitis, biliary obstruction, gastric outlet obstruction and rapid decline of hemoglobin after an endoscopic examination.

In uncomplicated cases, IDH usually resolves spontaneously with conservative treatment in 1–3 weeks [15]. Sadio *et al.* have reported a case in a patient with intramural gastric hematoma after endoscopic injection therapy, and have found that patient’s hematoma disappear six days later [14]. In our patient, the IDH was spontaneously resolved under abdominal sonography six days after the endoscopy.

To our best knowledge, less than 10 cases of patients with esophageal pneumatosis have been reported [4, 16], and no esophagogastroduodenal pneumatosis has been reported. The mechanism of PI has been experimentally proved that dissection of the gas from intraluminal to the intramural compartment is due to increased intra-abdominal pressure combined with mucosal defect [1]. We hypothesize that the findings of our patient are derived from the similar mechanism. The air was inflated from the defect of the needle injection site after endoscopic hemostasis for duodenal ulcer hemorrhage, and warfarin-associated coagulopathy precipitated the formation of IDH, which further dissected the duodenal wall, leading to inflated air entering into the duodenal, gastric and esophageal wall. The inflated air finally entered the portal vein through the portal circulation system.

Traditionally, once the pneumoporta is recognized along with PI, surgical intervention is preferred due to the concern of having ischemic bowel. But, PI is a radiological finding with wide spectrum of clinical severity and outcome. Surgical or conservative treatment should be considered according to the underlying etiology [1]. In this patient, esophagogastroduodenal pneumatosis and intramural duodenal hematoma were resolved spontaneously six days later. Therefore, we suggest that conservative treatment and close clinical assessment should be considered as an initial management for patients who complicate with IDH and PI after an endoscopic procedure. But, surgery is needed for those patients if the diseases progress or complications persist.

## Conclusion

Intramural hematoma and PI may be an adverse effect after endoscopic intervention, especially in patients with coagulopathy. The patient with the tetrad symptoms of acute pancreatitis, biliary obstruction, gastric outlet obstruction and rapid decline of hemoglobin after duodenal endoscopic intervention should be evaluated for the IDH. We suggest that those patients might be treated with conservative care initially, but surgical intervention is needed if the diseases progress or complications persist.

### Consent

Written informed consent was obtained from the patient’s wife for publication of this case report and any accompanying images. A copy of the written consent is available for review by the editors of this journal.

## Abbreviations

PI: Pneumatosis intestinalis
IDH: Intramural duodenal hematoma
PT: Prothrombin time
aPTT: activated partial thromboplastin time
ERCP: Endoscopic retrograde cholangiopancreatogram
